# Terbium (lithium zinc) distannide, TbLi_1–*x*_Zn_*x*_Sn_2_ (*x* = 0.2)

**DOI:** 10.1107/S1600536812002103

**Published:** 2012-01-21

**Authors:** Andrij Stetskiv, Ivan Tarasiuk, Beata Rozdzynska-Kielbik, Igor Oshchapovsky, Volodymyr Pavlyuk

**Affiliations:** a Ivano-Frankivsk National Medical University, Department of Chemistry, Galytska str. 2, 76018 Ivano-Frankivsk, Ukraine; b Department of Inorganic Chemistry, Ivan Franko Lviv National University, Kyryla and Mefodiya str. 6, 79005 Lviv, Ukraine; c Institute of Chemistry, Environment Protection and Biotechnology, Jan Dlugosz University, al. Armii Krajowej 13/15, 42-200 Czestochowa, Poland

## Abstract

The new terbium (lithium zinc) distannide, TbLi_1–*x*_Zn_*x*_Sn_2_ (*x* = 0.2) crystallizes in the ortho­rhom­bic CeNiSi_2_ structure type with space group *Cmcm* and Pearson symbol *oS*16. Of the four independent 4*c* atom positions (*m*2*m* site symmetry), three are fully occupied by individual atoms (two by Sn and one by Tb atoms) and the fourth is occupied by Li and Zn atoms with a statistical distribution. The Tb coordination polyhedron is a 21-vertex pseudo-Frank–Kasper polyhedron. One Sn atom is enclosed in a tricapped trigonal prism, the second Sn atom is in a cubocta­hedron and the statistically distributed (Li,Zn) site is in a tetra­gonal anti­prism with one added atom. Electronic structure calculations were used for the elucidation of reasons for and the ability of mutual substitution of lithium and transition metals. Positive charge density was observed around the rare earth atom and the Li and Zn atoms, the negative charge density in the proximity of the Sn atoms.

## Related literature

For general background, see: Andersen *et al.* (1986 ▶); Pavlyuk *et al.* (2009 ▶). For related structures, see: Pavlyuk & Bodak (1992*a* ▶,*b* ▶); Pavlyuk *et al.* (1991 ▶, 1993 ▶). For isotypic structures, see: Pavlyuk *et al.* (1989 ▶).

## Experimental

### 

#### Crystal data

TbLi_0.8_Zn_0.2_Sn_2_
*M* *_r_* = 414.85
Orthorhombic, 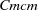

*a* = 4.4495 (7) Å
*b* = 17.699 (3) Å
*c* = 4.3978 (7) Å
*V* = 346.33 (9) Å^3^
*Z* = 4
Mo *K*α radiation
μ = 35.55 mm^−1^
*T* = 293 K
0.08 × 0.04 × 0.02 mm

#### Data collection

Oxford Diffraction Xcalibur3 CCD diffractometer
Absorption correction: analytical (*CrysAlis RED*; Oxford Diffraction, 2008 ▶) *T*_min_ = 0.344, *T*_max_ = 0.658
1198 measured reflections
261 independent reflections
190 reflections with *I* > 2σ(*I*)
*R*_int_ = 0.041

#### Refinement

*R*[*F* ^2^ > 2σ(*F* ^2^)] = 0.027
*wR*(*F* ^2^) = 0.066
*S* = 1.19
261 reflections
20 parameters
Δρ_max_ = 2.15 e Å^−3^
Δρ_min_ = −2.64 e Å^−3^

### 

Data collection: *CrysAlis CCD* (Oxford Diffraction, 2008 ▶); cell refinement: *CrysAlis CCD*; data reduction: *CrysAlis RED* (Oxford Diffraction, 2008 ▶); program(s) used to solve structure: *SHELXS97* (Sheldrick, 2008 ▶); program(s) used to refine structure: *SHELXL97* (Sheldrick, 2008 ▶); molecular graphics: *DIAMOND* (Brandenburg, 2006 ▶); software used to prepare material for publication: *SHELXL97*.

## Supplementary Material

Crystal structure: contains datablock(s) I, global. DOI: 10.1107/S1600536812002103/fi2120sup1.cif

Structure factors: contains datablock(s) I. DOI: 10.1107/S1600536812002103/fi2120Isup2.hkl

Additional supplementary materials:  crystallographic information; 3D view; checkCIF report

## Figures and Tables

**Fig. 1.**
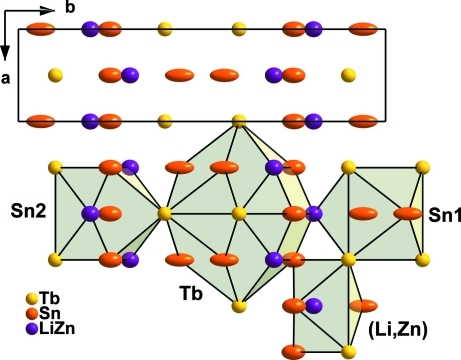
Unit cell projection and coordination polyhedra of atoms in the TbLi1–xZnxSn2 compound. Thermal ellipsoids are drawn at a 95% probability level.

**Fig. 2.**
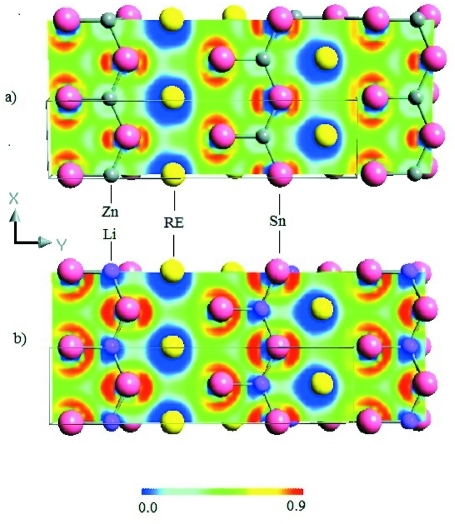
The results of electron localization function calculations for ordered structure models of REZnSn2 (a) and RELiSn2 (b). ELF map drawn at z = 0.

## supplementary crystallographic information

### Comment

During the systematic investigation of alloys of the Tb–Li–Sn and Tb–Zn–Sn ternary systems the ternary compounds with the corresponding compositions TbLiSn_2_ (Pavlyuk *et al.*, 1989) and TbZnSn_2_ (Pavlyuk *et al.*, 2009) were found. According to the X-ray data, TbLiSn_2_ crystallizes with orthorhombic symmetry (space group *Cmcm*, CeNiSi_2_ structure type) and the TbZnSn_2_ with tetragonal symmetry (space group *P*4/*nmm*, HfCuSi_2_ structure type). Structural studies of the four-component alloys from TbLiSn_2_–TbZnSn_2_ sections indicate the existence of TbLi_1–_*_x_*Zn*_x_*Sn_2_ (*x* = 0 - 1/5) limited solid solution. In the ternary TbLiSn_2_ compound lithium atoms occupy the same crystallographic position that the atoms of transition metal in the original CeNiSi_2_ structure type. The same was observed previously when we studied RELiGe with the ZrNiAl type (Pavlyuk *et al.*, 1991 and Pavlyuk *et al.*, 1992*a*) and RE_3_Li_2_Ge_3_ with Hf_3_Ni_2_Si_3_ type (Pavlyuk & Bodak, 1992*b*). X-ray single-crystal study showed that the TbLi_1–_*_x_*Zn*_x_*Sn_2_ solid solution formed by the partial substitution of lithium atoms by zinc atoms in 4*c* site. The ability of lithium atoms to substitute the atoms of transition metals we observed previously studying solid solutions RELi*_x_*Cu_2–_*_x_*Si_2_ and RELi*_x_*Cu_2–_*_x_*Ge_2_ (Pavlyuk *et al.*, 1993).

The projection of the unit cell and coordination polyhedra of the atoms are shown in Fig. 1. The coordination polyhedra of atoms are: Tb1 – 21-vertex pseudo Frank-Kasper polyhedron [Tb(Zn,Li)_5_Sn_10_Tb_6_], Sn1 – 9-vertex tricapped trigonal prism [Sn1(Li,Zn)Sn_2_Tb_6_], Sn2 – 12-vertex cuboctahedron [Sn2(Li,Zn)_4_Sn_4_Tb_4_] and statistical mixture (Li,Zn) – 9-vertex tetragonal antiprism with one added atom [(Li,Zn)Sn_5_Tb_4_].

Formation of the same RELi_1–_*_x_*Zn*_x_*Sn_2_ solid solutions were observed with other rare earth metals, such as Gd, Dy, Ho and Y.

The electronic structure calculations using TB-LMTO-ASA (Andersen *et al.*, 1986) program package were performed for the elucidation of reasons of the formation of solid solutions and the ability to mutual substitution of lithium and transition metals. The ordered model of RELiSn_2_ ternary phase and hypothetical REZnSn_2_ with CeNiSi_2_ structure type were analyzed. Among the rare earth metals was taken yttrium, which has less number of electrons than majority of other rare earth metals.

According to the results of calculations in the both models the rare earth atoms donate their electrons to tin atoms. The lithium atoms (Fig. 2a) and zinc atoms (Fig. 2 b) also loses their electrons. So positive charge density in various scale can be observed around rare earth, lithium and zinc atoms and negative charge density is around tin atoms. Taking into account these data and also the closeness of the effective radius of zinc and lithium atoms in intermetallic compounds it can be concluded that nothing prevents their mutual substitution.

### Experimental

Terbium, lithium, zinc and tin, all with a nominal purity more than 99.9 wt. %, were used as starting elements. First, the pieces of the pure metals with a stoichiometry Tb_25_Li_20_Zn_5_Sn_50_ were pressed into pellets, enclosed in a tantalum crucible and placed in a resistance furnace with a thermocouple controller. The sample was heated to 670 K at a rate of 5 K/min, maintained over a period of 48 h and then temperature was increased to 1070 K over a period of 10 h. The alloy was annealed at 670 K for 120 h and cooled slowly to room temperature. Small, good-quality single-crystals of the title compound were isolated from an alloy by mechanical fragmentation.

### Refinement

The Li position (Wyckoff sites 4*c*) showed displacement parameters considerably smaller than it should be for the lithium, suggesting that this position is partially occupied by the heavier Zn atom. The refinement of the occupancy of this statistically mixed position showed, that it contains 80% of Li and 20% of Zn atoms.

### Crystal data

**Table:**

TbLi_0.8_Zn_0.2_Sn_2_	*F*(000) = 693.6
*M**_r_* = 414.85	*D*_x_ = 7.956 Mg m^−^^3^
Orthorhombic, *C**m**c**m*	Mo *K*α radiation, λ = 0.71073 Å
Hall symbol: -C 2c 2	Cell parameters from 261 reflections
*a* = 4.4495 (7) Å	θ = 4.6–28.0°
*b* = 17.699 (3) Å	µ = 35.55 mm^−^^1^
*c* = 4.3978 (7) Å	*T* = 293 K
*V* = 346.33 (9) Å^3^	Prism, metallic dark grey
*Z* = 4	0.08 × 0.04 × 0.02 mm

### Data collection

**Table:**

Oxford Diffraction Xcalibur3 CCD diffractometer	261 independent reflections
Radiation source: fine-focus sealed tube	190 reflections with *I* > 2σ(*I*)
graphite	*R*_int_ = 0.041
Detector resolution: 0 pixels mm^-1^	θ_max_ = 28.0°, θ_min_ = 4.6°
ω scans	*h* = −5→5
Absorption correction: analytical (*CrysAlis RED*; Oxford Diffraction, 2008)	*k* = −17→23
*T*_min_ = 0.344, *T*_max_ = 0.658	*l* = −5→5
1198 measured reflections	

### Refinement

**Table:**

Refinement on *F*^2^	Primary atom site location: structure-invariant direct methods
Least-squares matrix: full	Secondary atom site location: difference Fourier map
*R*[*F*^2^ > 2σ(*F*^2^)] = 0.027	*w* = 1/[σ^2^(*F*_o_^2^) + (0.0272*P*)^2^ + 2.6711*P*] where *P* = (*F*_o_^2^ + 2*F*_c_^2^)/3
*wR*(*F*^2^) = 0.066	(Δ/σ)_max_ < 0.001
*S* = 1.19	Δρ_max_ = 2.15 e Å^−^^3^
261 reflections	Δρ_min_ = −2.64 e Å^−^^3^
20 parameters	Extinction correction: *SHELXL97* (Sheldrick, 2008), Fc^*^=kFc[1+0.001xFc^2^λ^3^/sin(2θ)]^-1/4^
0 restraints	Extinction coefficient: 0.0039 (4)

### Special details

**Table:**

Geometry. All e.s.d.'s (except the e.s.d. in the dihedral angle between two l.s. planes) are estimated using the full covariance matrix. The cell e.s.d.'s are taken into account individually in the estimation of e.s.d.'s in distances, angles and torsion angles; correlations between e.s.d.'s in cell parameters are only used when they are defined by crystal symmetry. An approximate (isotropic) treatment of cell e.s.d.'s is used for estimating e.s.d.'s involving l.s. planes.
Refinement. Refinement of *F*^2^ against ALL reflections. The weighted *R*-factor *wR* and goodness of fit *S* are based on *F*^2^, conventional *R*-factors *R* are based on *F*, with *F* set to zero for negative *F*^2^. The threshold expression of *F*^2^ > σ(*F*^2^) is used only for calculating *R*-factors(gt) *etc*. and is not relevant to the choice of reflections for refinement. *R*-factors based on *F*^2^ are statistically about twice as large as those based on *F*, and *R*- factors based on ALL data will be even larger.

### Fractional atomic coordinates and isotropic or equivalent isotropic displacement parameters (Å^2^)

**Table:**

	*x*	*y*	*z*	*U*_iso_*/*U*_eq_	Occ. (<1)
Tb1	0.0000	0.39911 (5)	0.2500	0.0156 (4)	
Sn1	0.0000	0.06101 (10)	0.2500	0.0296 (6)	
Sn2	0.0000	0.75076 (8)	0.2500	0.0274 (6)	
Zn	0.0000	0.1960 (5)	0.2500	0.022 (3)	0.198 (11)
Li	0.0000	0.1960 (5)	0.2500	0.022 (3)	0.80

### Atomic displacement parameters (Å^2^)

**Table:**

	*U*^11^	*U*^22^	*U*^33^	*U*^12^	*U*^13^	*U*^23^
Tb1	0.0152 (5)	0.0167 (5)	0.0149 (6)	0.000	0.000	0.000
Sn1	0.0132 (8)	0.0638 (16)	0.0119 (9)	0.000	0.000	0.000
Sn2	0.0180 (8)	0.0423 (14)	0.0220 (10)	0.000	0.000	0.000
Zn	0.019 (5)	0.024 (5)	0.022 (6)	0.000	0.000	0.000
Li	0.019 (5)	0.024 (5)	0.022 (6)	0.000	0.000	0.000

### Geometric parameters (Å, °)

**Table:**

Tb1—Sn1^i^	3.2067 (6)	Sn2—Li^viii^	2.392 (4)
Tb1—Sn1^ii^	3.2067 (6)	Sn2—Zn^viii^	2.392 (4)
Tb1—Sn1^iii^	3.2067 (6)	Sn2—Li^vii^	2.392 (4)
Tb1—Sn1^iv^	3.2067 (6)	Sn2—Zn^vii^	2.392 (4)
Tb1—Sn2^v^	3.4414 (13)	Sn2—Li^xi^	2.427 (4)
Tb1—Sn2^vi^	3.4414 (13)	Sn2—Zn^xi^	2.427 (4)
Tb1—Sn2^vii^	3.4454 (14)	Sn2—Li^xii^	2.427 (4)
Tb1—Sn2^viii^	3.4454 (14)	Sn2—Zn^xii^	2.427 (4)
Tb1—Li^ii^	3.552 (4)	Sn2—Sn2^xiii^	3.1282 (4)
Tb1—Zn^ii^	3.552 (4)	Sn2—Sn2^xiv^	3.1282 (4)
Tb1—Li^i^	3.552 (4)	Sn2—Sn2^xv^	3.1282 (4)
Tb1—Zn^i^	3.552 (4)	Sn2—Sn2^xvi^	3.1282 (4)
Sn1—Zn	2.389 (9)	Zn—Sn2^viii^	2.392 (4)
Sn1—Sn1^ix^	3.082 (3)	Zn—Sn2^vii^	2.392 (4)
Sn1—Sn1^x^	3.082 (3)	Zn—Sn2^vi^	2.427 (4)
Sn1—Tb1^i^	3.2067 (6)	Zn—Sn2^v^	2.427 (4)
Sn1—Tb1^ii^	3.2067 (6)	Zn—Tb1^ii^	3.552 (4)
Sn1—Tb1^iv^	3.2067 (6)	Zn—Tb1^i^	3.552 (4)
Sn1—Tb1^iii^	3.2067 (6)	Zn—Tb1^iii^	3.552 (4)
Sn1—Tb1^v^	3.6277 (15)	Zn—Tb1^iv^	3.552 (4)
Sn1—Tb1^vi^	3.6277 (15)		
			
Sn1^i^—Tb1—Sn1^ii^	154.57 (8)	Li^viii^—Sn2—Zn^viii^	0.0
Sn1^i^—Tb1—Sn1^iii^	87.86 (2)	Li^viii^—Sn2—Li^vii^	133.6 (4)
Sn1^ii^—Tb1—Sn1^iii^	86.59 (2)	Zn^viii^—Sn2—Li^vii^	133.6 (4)
Sn1^i^—Tb1—Sn1^iv^	86.59 (2)	Li^viii^—Sn2—Zn^vii^	133.6 (4)
Sn1^ii^—Tb1—Sn1^iv^	87.86 (2)	Zn^viii^—Sn2—Zn^vii^	133.6 (4)
Sn1^iii^—Tb1—Sn1^iv^	154.57 (8)	Li^vii^—Sn2—Zn^vii^	0.0 (4)
Sn1^i^—Tb1—Sn2^v^	128.06 (4)	Li^viii^—Sn2—Li^xi^	99.05 (15)
Sn1^ii^—Tb1—Sn2^v^	73.71 (3)	Zn^viii^—Sn2—Li^xi^	99.05 (15)
Sn1^iii^—Tb1—Sn2^v^	73.71 (3)	Li^vii^—Sn2—Li^xi^	99.05 (15)
Sn1^iv^—Tb1—Sn2^v^	128.06 (4)	Zn^vii^—Sn2—Li^xi^	99.05 (15)
Sn1^i^—Tb1—Sn2^vi^	73.71 (3)	Li^viii^—Sn2—Zn^xi^	99.05 (15)
Sn1^ii^—Tb1—Sn2^vi^	128.06 (4)	Zn^viii^—Sn2—Zn^xi^	99.05 (15)
Sn1^iii^—Tb1—Sn2^vi^	128.06 (4)	Li^vii^—Sn2—Zn^xi^	99.05 (15)
Sn1^iv^—Tb1—Sn2^vi^	73.71 (3)	Zn^vii^—Sn2—Zn^xi^	99.05 (15)
Sn2^v^—Tb1—Sn2^vi^	80.55 (4)	Li^xi^—Sn2—Zn^xi^	0.0
Sn1^i^—Tb1—Sn2^vii^	127.38 (4)	Li^viii^—Sn2—Li^xii^	99.05 (15)
Sn1^ii^—Tb1—Sn2^vii^	74.44 (3)	Zn^viii^—Sn2—Li^xii^	99.05 (15)
Sn1^iii^—Tb1—Sn2^vii^	127.38 (4)	Li^vii^—Sn2—Li^xii^	99.05 (15)
Sn1^iv^—Tb1—Sn2^vii^	74.44 (3)	Zn^vii^—Sn2—Li^xii^	99.05 (15)
Sn2^v^—Tb1—Sn2^vii^	54.030 (13)	Li^xi^—Sn2—Li^xii^	132.9 (4)
Sn2^vi^—Tb1—Sn2^vii^	54.030 (13)	Zn^xi^—Sn2—Li^xii^	132.9 (4)
Sn1^i^—Tb1—Sn2^viii^	74.44 (3)	Li^viii^—Sn2—Zn^xii^	99.05 (15)
Sn1^ii^—Tb1—Sn2^viii^	127.38 (4)	Zn^viii^—Sn2—Zn^xii^	99.05 (15)
Sn1^iii^—Tb1—Sn2^viii^	74.44 (3)	Li^vii^—Sn2—Zn^xii^	99.05 (15)
Sn1^iv^—Tb1—Sn2^viii^	127.38 (4)	Zn^vii^—Sn2—Zn^xii^	99.05 (15)
Sn2^v^—Tb1—Sn2^viii^	54.030 (13)	Li^xi^—Sn2—Zn^xii^	132.9 (4)
Sn2^vi^—Tb1—Sn2^viii^	54.030 (13)	Zn^xi^—Sn2—Zn^xii^	132.9 (4)
Sn2^vii^—Tb1—Sn2^viii^	79.32 (4)	Li^xii^—Sn2—Zn^xii^	0.0 (4)
Sn1^i^—Tb1—Li^ii^	164.43 (14)	Li^viii^—Sn2—Sn2^xiii^	130.50 (8)
Sn1^ii^—Tb1—Li^ii^	41.00 (13)	Zn^viii^—Sn2—Sn2^xiii^	130.50 (8)
Sn1^iii^—Tb1—Li^ii^	95.41 (3)	Li^vii^—Sn2—Sn2^xiii^	50.01 (8)
Sn1^iv^—Tb1—Li^ii^	96.57 (3)	Zn^vii^—Sn2—Sn2^xiii^	50.01 (8)
Sn2^v^—Tb1—Li^ii^	39.97 (9)	Li^xi^—Sn2—Sn2^xiii^	49.05 (7)
Sn2^vi^—Tb1—Li^ii^	92.49 (12)	Zn^xi^—Sn2—Sn2^xiii^	49.05 (7)
Sn2^vii^—Tb1—Li^ii^	40.55 (9)	Li^xii^—Sn2—Sn2^xiii^	130.43 (9)
Sn2^viii^—Tb1—Li^ii^	91.74 (12)	Zn^xii^—Sn2—Sn2^xiii^	130.43 (9)
Sn1^i^—Tb1—Zn^ii^	164.43 (14)	Li^viii^—Sn2—Sn2^xiv^	50.01 (8)
Sn1^ii^—Tb1—Zn^ii^	41.00 (13)	Zn^viii^—Sn2—Sn2^xiv^	50.01 (8)
Sn1^iii^—Tb1—Zn^ii^	95.41 (3)	Li^vii^—Sn2—Sn2^xiv^	130.50 (8)
Sn1^iv^—Tb1—Zn^ii^	96.57 (3)	Zn^vii^—Sn2—Sn2^xiv^	130.50 (8)
Sn2^v^—Tb1—Zn^ii^	39.97 (9)	Li^xi^—Sn2—Sn2^xiv^	130.43 (9)
Sn2^vi^—Tb1—Zn^ii^	92.49 (12)	Zn^xi^—Sn2—Sn2^xiv^	130.43 (9)
Sn2^vii^—Tb1—Zn^ii^	40.55 (9)	Li^xii^—Sn2—Sn2^xiv^	49.05 (7)
Sn2^viii^—Tb1—Zn^ii^	91.74 (12)	Zn^xii^—Sn2—Sn2^xiv^	49.05 (7)
Li^ii^—Tb1—Zn^ii^	0.0 (3)	Sn2^xiii^—Sn2—Sn2^xiv^	179.01 (11)
Sn1^i^—Tb1—Li^i^	41.00 (13)	Li^viii^—Sn2—Sn2^xv^	50.01 (8)
Sn1^ii^—Tb1—Li^i^	164.43 (14)	Zn^viii^—Sn2—Sn2^xv^	50.01 (8)
Sn1^iii^—Tb1—Li^i^	96.57 (3)	Li^vii^—Sn2—Sn2^xv^	130.50 (8)
Sn1^iv^—Tb1—Li^i^	95.41 (3)	Zn^vii^—Sn2—Sn2^xv^	130.50 (8)
Sn2^v^—Tb1—Li^i^	92.49 (12)	Li^xi^—Sn2—Sn2^xv^	49.05 (7)
Sn2^vi^—Tb1—Li^i^	39.97 (9)	Zn^xi^—Sn2—Sn2^xv^	49.05 (7)
Sn2^vii^—Tb1—Li^i^	91.74 (12)	Li^xii^—Sn2—Sn2^xv^	130.43 (9)
Sn2^viii^—Tb1—Li^i^	40.55 (9)	Zn^xii^—Sn2—Sn2^xv^	130.43 (9)
Li^ii^—Tb1—Li^i^	123.4 (3)	Sn2^xiii^—Sn2—Sn2^xv^	89.326 (13)
Zn^ii^—Tb1—Li^i^	123.4 (3)	Sn2^xiv^—Sn2—Sn2^xv^	90.665 (13)
Sn1^i^—Tb1—Zn^i^	41.00 (13)	Li^viii^—Sn2—Sn2^xvi^	130.50 (8)
Sn1^ii^—Tb1—Zn^i^	164.43 (14)	Zn^viii^—Sn2—Sn2^xvi^	130.50 (8)
Sn1^iii^—Tb1—Zn^i^	96.57 (3)	Li^vii^—Sn2—Sn2^xvi^	50.01 (8)
Sn1^iv^—Tb1—Zn^i^	95.41 (3)	Zn^vii^—Sn2—Sn2^xvi^	50.01 (8)
Sn2^v^—Tb1—Zn^i^	92.49 (12)	Li^xi^—Sn2—Sn2^xvi^	130.43 (9)
Sn2^vi^—Tb1—Zn^i^	39.97 (9)	Zn^xi^—Sn2—Sn2^xvi^	130.43 (9)
Sn2^vii^—Tb1—Zn^i^	91.74 (12)	Li^xii^—Sn2—Sn2^xvi^	49.05 (7)
Sn2^viii^—Tb1—Zn^i^	40.55 (9)	Zn^xii^—Sn2—Sn2^xvi^	49.05 (7)
Li^ii^—Tb1—Zn^i^	123.4 (3)	Sn2^xiii^—Sn2—Sn2^xvi^	90.665 (13)
Zn^ii^—Tb1—Zn^i^	123.4 (3)	Sn2^xiv^—Sn2—Sn2^xvi^	89.326 (13)
Li^i^—Tb1—Zn^i^	0.0 (3)	Sn2^xv^—Sn2—Sn2^xvi^	179.01 (11)
Zn—Sn1—Sn1^ix^	134.48 (5)	Sn1—Zn—Sn2^viii^	113.2 (2)
Zn—Sn1—Sn1^x^	134.48 (5)	Sn1—Zn—Sn2^vii^	113.2 (2)
Sn1^ix^—Sn1—Sn1^x^	91.03 (10)	Sn2^viii^—Zn—Sn2^vii^	133.6 (4)
Zn—Sn1—Tb1^i^	77.28 (4)	Sn1—Zn—Sn2^vi^	113.5 (2)
Sn1^ix^—Sn1—Tb1^i^	130.05 (5)	Sn2^viii^—Zn—Sn2^vi^	80.95 (15)
Sn1^x^—Sn1—Tb1^i^	70.427 (15)	Sn2^vii^—Zn—Sn2^vi^	80.95 (15)
Zn—Sn1—Tb1^ii^	77.28 (4)	Sn1—Zn—Sn2^v^	113.5 (2)
Sn1^ix^—Sn1—Tb1^ii^	70.427 (15)	Sn2^viii^—Zn—Sn2^v^	80.95 (15)
Sn1^x^—Sn1—Tb1^ii^	130.05 (5)	Sn2^vii^—Zn—Sn2^v^	80.95 (15)
Tb1^i^—Sn1—Tb1^ii^	154.57 (8)	Sn2^vi^—Zn—Sn2^v^	132.9 (4)
Zn—Sn1—Tb1^iv^	77.28 (4)	Sn1—Zn—Tb1^ii^	61.72 (13)
Sn1^ix^—Sn1—Tb1^iv^	70.427 (15)	Sn2^viii^—Zn—Tb1^ii^	139.08 (5)
Sn1^x^—Sn1—Tb1^iv^	130.05 (5)	Sn2^vii^—Zn—Tb1^ii^	67.52 (6)
Tb1^i^—Sn1—Tb1^iv^	86.59 (2)	Sn2^vi^—Zn—Tb1^ii^	139.77 (5)
Tb1^ii^—Sn1—Tb1^iv^	87.86 (2)	Sn2^v^—Zn—Tb1^ii^	67.36 (6)
Zn—Sn1—Tb1^iii^	77.28 (4)	Sn1—Zn—Tb1^i^	61.72 (13)
Sn1^ix^—Sn1—Tb1^iii^	130.05 (5)	Sn2^viii^—Zn—Tb1^i^	67.52 (6)
Sn1^x^—Sn1—Tb1^iii^	70.427 (15)	Sn2^vii^—Zn—Tb1^i^	139.08 (5)
Tb1^i^—Sn1—Tb1^iii^	87.86 (2)	Sn2^vi^—Zn—Tb1^i^	67.36 (6)
Tb1^ii^—Sn1—Tb1^iii^	86.59 (2)	Sn2^v^—Zn—Tb1^i^	139.77 (5)
Tb1^iv^—Sn1—Tb1^iii^	154.57 (8)	Tb1^ii^—Zn—Tb1^i^	123.4 (3)
Zn—Sn1—Tb1^v^	142.173 (19)	Sn1—Zn—Tb1^iii^	61.72 (13)
Sn1^ix^—Sn1—Tb1^v^	56.40 (4)	Sn2^viii^—Zn—Tb1^iii^	67.52 (6)
Sn1^x^—Sn1—Tb1^v^	56.40 (4)	Sn2^vii^—Zn—Tb1^iii^	139.08 (5)
Tb1^i^—Sn1—Tb1^v^	126.82 (4)	Sn2^vi^—Zn—Tb1^iii^	139.77 (5)
Tb1^ii^—Sn1—Tb1^v^	75.43 (3)	Sn2^v^—Zn—Tb1^iii^	67.36 (6)
Tb1^iv^—Sn1—Tb1^v^	126.82 (4)	Tb1^ii^—Zn—Tb1^iii^	76.49 (11)
Tb1^iii^—Sn1—Tb1^v^	75.43 (3)	Tb1^i^—Zn—Tb1^iii^	77.56 (11)
Zn—Sn1—Tb1^vi^	142.173 (19)	Sn1—Zn—Tb1^iv^	61.72 (13)
Sn1^ix^—Sn1—Tb1^vi^	56.40 (4)	Sn2^viii^—Zn—Tb1^iv^	139.08 (5)
Sn1^x^—Sn1—Tb1^vi^	56.40 (4)	Sn2^vii^—Zn—Tb1^iv^	67.52 (6)
Tb1^i^—Sn1—Tb1^vi^	75.43 (3)	Sn2^vi^—Zn—Tb1^iv^	67.36 (6)
Tb1^ii^—Sn1—Tb1^vi^	126.82 (4)	Sn2^v^—Zn—Tb1^iv^	139.77 (5)
Tb1^iv^—Sn1—Tb1^vi^	75.43 (3)	Tb1^ii^—Zn—Tb1^iv^	77.56 (11)
Tb1^iii^—Sn1—Tb1^vi^	126.82 (4)	Tb1^i^—Zn—Tb1^iv^	76.49 (11)
Tb1^v^—Sn1—Tb1^vi^	75.65 (4)	Tb1^iii^—Zn—Tb1^iv^	123.4 (3)

Symmetry codes: (i) −*x*+1/2, −*y*+1/2, −*z*+1; (ii) −*x*−1/2, −*y*+1/2, −*z*; (iii) −*x*−1/2, −*y*+1/2, −*z*+1; (iv) −*x*+1/2, −*y*+1/2, −*z*; (v) *x*−1/2, *y*−1/2, *z*; (vi) *x*+1/2, *y*−1/2, *z*; (vii) −*x*, −*y*+1, −*z*; (viii) −*x*, −*y*+1, −*z*+1; (ix) −*x*, −*y*, −*z*; (x) −*x*, −*y*, −*z*+1; (xi) *x*−1/2, *y*+1/2, *z*; (xii) *x*+1/2, *y*+1/2, *z*; (xiii) −*x*−1/2, −*y*+3/2, −*z*; (xiv) −*x*+1/2, −*y*+3/2, −*z*+1; (xv) −*x*−1/2, −*y*+3/2, −*z*+1; (xvi) −*x*+1/2, −*y*+3/2, −*z*.
